# Bacterial community structure upstream and downstream of cascade dams along the Lancang River in southwestern China

**DOI:** 10.1007/s11356-020-10159-7

**Published:** 2020-07-28

**Authors:** Xia Luo, Xinyi Xiang, Guoyi Huang, Xiaorui Song, Peijia Wang, Yuanhao Yang, Kaidao Fu, Rongxiao Che

**Affiliations:** 1 Institute of International Rivers and Eco-Security, Yunnan University, Chenggong University Town, Chenggong New District, Kunming, 650500 Yunnan Province China; 2 Yunnan Key Laboratory of International Rivers and Transboundary Eco-Security, Kunming, 650500 China

**Keywords:** Bacterial community, Water, Sediment, Spatiotemporal distribution, Cascade dam construction, Lancang River, Responsible Editor: Diane Purchase

## Abstract

Extensive construction of dams by humans has caused alterations in flow regimes and concomitant alterations in river ecosystems. Even so, bacterioplankton diversity in large rivers influenced by cascade dams has been largely ignored. In this study, bacterial community diversity and profiles of seven cascade dams along the720 km of the Lancang River were studied using Illumina sequencing of the V3-V4 hypervariable region of the 16S rRNA gene. Spatiotemporal variations of bacterial communities in sediment and water of the Gongguoqiao hydroelectric dam and factors affecting these variations were also examined. Microbial diversity and richness in surface water increased slightly from upstream toward downstream along the river. A significant positive correlation between spatial distance and dissimilarities in bacterial community structure was confirmed (Mantel test, *r* = 0.4826, *p* = 0.001). At the Gongguoqiao hydroelectric dam, temporal differences in water overwhelmed spatial variability in bacterial communities. Temperature, precipitation, and nutrient levels were major drivers of seasonal microbial changes. Most functional groups associated with carbon cycling in sediment samples decreased from winter to summer. Our findings improve our understanding of associations, compositions, and predicted functional profiles of microbial communities in a large riverine ecosystem influenced by multiple cascade dams.

**Electronic supplementary material:**

The online version of this article (10.1007/s11356-020-10159-7) contains supplementary material, which is available to authorized users.

## Introduction

Bacteria are crucial to biogeochemical processes in all aquatic ecosystems (Feng et al. 2009; Sekiguchi et al. 2002). These microprobes dominate aquatic ecosystems in abundance, diversity and metabolic activity, and function in essential ecological processes, such as the remediation of polluted environments (Savio et al. 2015). Several recent studies indicate that bacteria may be sentinels of environmental change (Harnisz 2013; Wang et al. 2012b) given their sensitivity to changing conditions (Savio et al. 2015).

Typically, dam construction will affect overall ecosystem function (Domingues et al. 2012) by decreasing microbial diversity altering microbial assemblages. Awareness regarding changes in microbial diversity and community composition caused by dam construction is increasing (Domingues et al. 2012; Gibbons et al. 2014; Li et al. 2013; Wu et al. 2013), yet our understanding of structural responses of sediment bacteria and bacterioplankton to cascade dam placement is still limited. High variability in physical and biogeochemical conditions makes studies in this area difficult. In contrast to a single dam, cascade dams cause extensive alterations in the structure and function of river ecosystems. Zhai et al. (2010) found that the cascade dams improved flood control and that the relationship between cascade dams and ecological impacts was non-linear. Ouyang et al. (2011) reported effects on the proportion of sand in sediments and accumulation of nutrients was cumulative and more pronounced than changes caused by single dams. These findings suggest that effects of dam cascades are more complex and difficult to predict. Liu et al. (2018) first investigated spatiotemporal patterns of water and sediment bacterial communities in the Yantze River, China, under the influence of two huge dams. They reported that bacterial taxa in sediment decreased significantly immediately downstream of both dams due to severe riverbed scouring. Chen et al. (2018) looked deeply into longitudinal distribution patterns of sediment microbes and their responses to cascade dam construction along the Lancang River, China. They found abundance, activity, and diversity of sediment bacteria were significantly lower in dam reservoirs than at sites immediately downstream of dams, but then increased gradually downstream. These contradictions highlight the difficulty and importance in the studying cascade dam-induced biogeographic distribution of microbial communities.

Damming is important for water resource utilization in large rivers, and dam construction leads to changes in river ecological integrity (Zhai et al. 2010), water quality (Domingues et al. 2012; Li et al. 2013), sediment discharge (Li et al. 2013), and flow regimes (Poff et al. 2007; Zhou et al. 2013). These changes, in turn, greatly affect environmentally sensitive organisms, such as bacteria in sediment and water. For example, water and sediment retention behind a dam affects nutrient concentration (Zhou et al. 2013), stoichiometry (Morais et al. 2009), and light availability (Barbosa et al. 2010), resulting alterations in community composition and ecosystem services provided by microbial communities. However, microbial community composition and diversity in water and sediment environments may be quite different, reflecting their distinct physicochemical dynamics. For instance, bacteria in pond sediments originate from sediment erosion and deposition processes, while river bacteria originate from surface runoff, precipitation, groundwater, soil, and lakes (Liu et al. 2018). Reduced sediment load and limited sources of sediments due to dam impoundments (Wang et al. 2012a) restrict movement of sediment-associated bacteria from upstream to downstream, resulting in significant shifts in community structure and diversity.

The Lancang-Mekong River is typically divided into two parts (i.e., the Upper Mekong River basin, the Lancang River, and the Lower Mekong River Basin). This river system is a crucially important international river in Southeast Asia. This river’s source is in the eastern Tibetan Plateau in China, and it passes through parts of China, Myanmar, Laos, Thailand, Cambodia, and Vietnam, with a total length of 4880 km and a drainage basin of approximately 795,000 km^2^ (Fan et al. 2015). The upper Lancang River Basin, located in the Yunnan Province in southwestern China, has a channel length of 1170 km and a drop in elevation of 1780 m at an average gradient of 0.15% (Li et al. 2019). Twenty-one cascade hydropower dams along the river are planned (Fan et al. 2015). Seven cascading hydropower dams were completed by the end of 2017 in the middle and lower reaches of the mainstream in Yunnan Province to meet increasing energy needs. Available studies, however, focus on relatively small river basins or a small number of hydroelectric dams. Spatial patterns of microbial communities may be more clearly manifest in large river basins due to a larger contrast in environmental conditions. Currently, little information is available on the spatiotemporal distribution of microbial communities in reaches of the Lancang River under that are influenced by cascade dams. The present study aims to help fill this gap by mapping spatial and temporal distributions of taxonomic diversity, and describing associations of modifications in diversity with dam construction. This information is essential for monitoring the ecosystem function and health.

Gene sequencing with 16S rRNA was used to gain insight into the effect of cascade dams on microbial communities and better understand interactions between surface water and sediment bacteria. Specifically, community composition, structure, and diversity in the surface water of seven cascade dam and non-dam control sites were investigated. Additionally, microbial communities in sediment and surface water upstream and downstream of one large dam among the seven cascade dams were also analyzed. We hypothesized that (1) cascade dams would display similar spatial patterns of planktonic bacterial diversity from upstream to downstream due to increased environmental homogeneity across geographically disjunct regions; (2) microbial community structure and diversity between water and sediment might respond differently to dam construction because of a dramatic decrease in sediment load downstream.

## Materials and methods

### Site description and sample collection

**Fig. 1:**
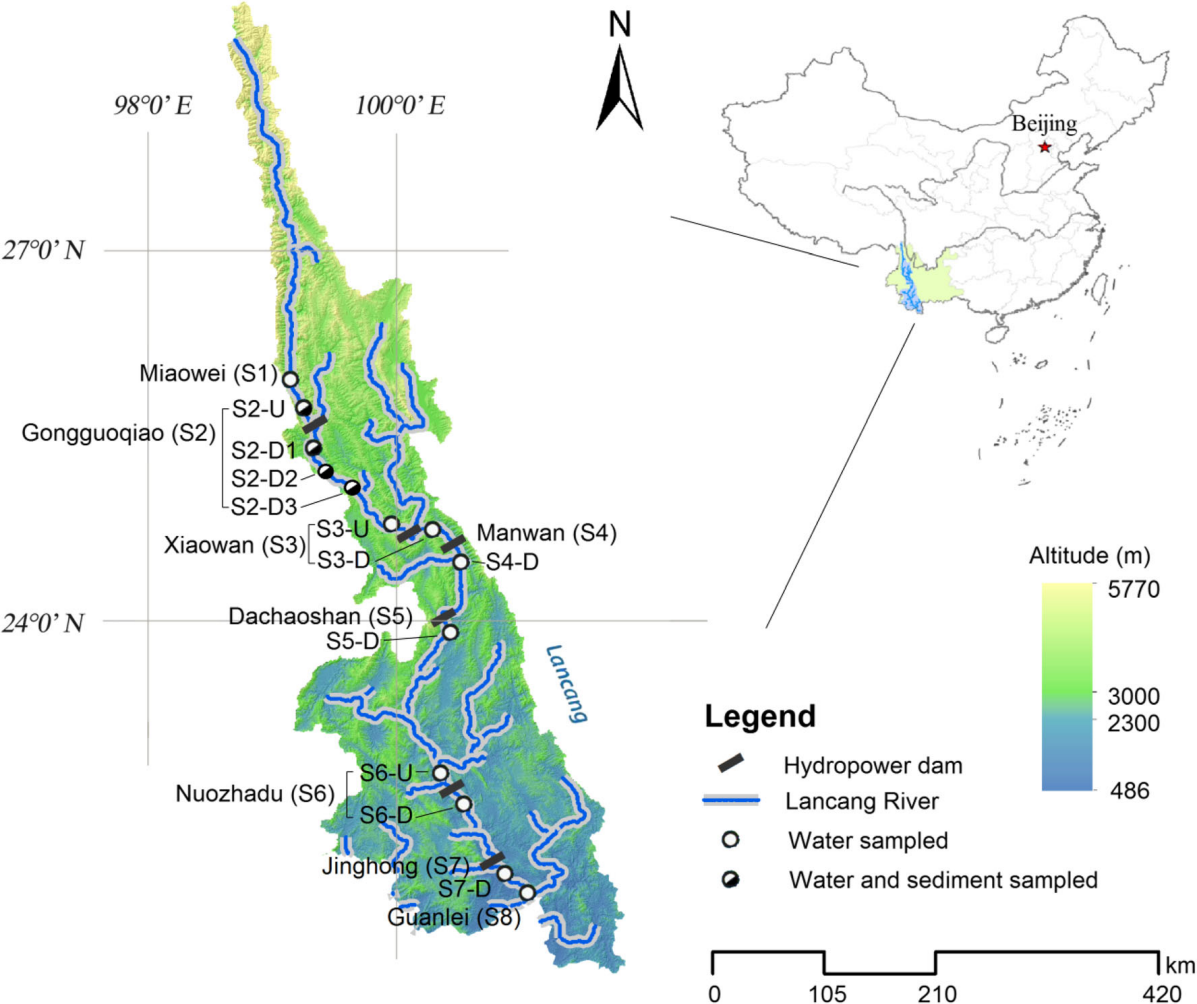
Overview and detailed map of the Lancang River basin and distribution of sampling sites across the river. The open dots represent the water sampling sites. The half-solid dots indicate the points where both the water and sediments were taken

Seven cascade hydropower dams in the middle and lower reaches of the mainstream in Yunnan Province, including Miaowei (S1), Gongguoqiao (S2), Xiaowan (S3), Manwan (S4), Dachaoshan (S5), Nuozhadu (S6), and Jinghong (S7) dams, have been built and put into use on the mainstream of Lancang-Mekong River, with the exception of Miaowei dam (S1). Details about these six operated dams are shown in Supplementary material Table S1. Eleven sampling sites with an elevation gradient from 487 to 1324 m extended over a length of approximately 728 km in this study. A total of 13 sampling sites along the mainstream of Lancang River from the downstream of Miaowei dam to the Guanlei station have been sampled (Fig. 1). Among those 13 sampling sites, S1 has not been put into operation, S8 locates at the China-Myanmar border where there is no dam planned. S2 is the first stage of the middle and lower reach of Lancang mainstream cascade development project, S2-U, S3-U, and S6-U locate at upstream of the dam (i.e., reservoir area behind the dam), S2-D1, S2-D2, S2-D3, S3-D, S4-D, S5-D, S6-D, and S7-D were below the hydropower dam (i.e., in front of the dam). Surface water (20 cm) samples from S1, S2-D1, S2-D2, S2-D3, S3-D, S4-D, S5-D, S6-D, S7-D, and S8, as well as sediment (10 cm) samples from S2-D1, S2-D2, S2-D3, and S3-D, were collected in January, 2017. To further investigate how seasonal variations and large dam construction affect bacterial communities, four representative sites: S2-U, S2-D1, S2-D2, and S2-D3 from upstream to downstream of Gongguoqiao dam were also sampled for surface water and sediments in July, 2017. Sediment samples were collected in duplicate (January, 2017) and triplicate (July, 2017) at approximately 33.1 ± 12.67 m of water depth in the reservoir (S2-U) using horizontal sediment sampler (inner diameter (ID) = 10.1 cm; tube length (L) = 45 cm), while clayey bank sediments at S2-D1, S2-D2, and S2-D3 were collected with a soil core sampler (ID = 38 mm; L = 50 cm). All sediment samples were homogenized and subsampled, and subsequently frozen in dry ice. Depend on biomass concentration, duplicate (January, 2017) or triplicate (July, 2017) 300–1500 mL of surface water samples was collected on 0.2 μm micropore membranes with a filter funnel and vacuum system. After filtration, the filters from each site were stored in dry ice. Sediments and filters were subsequently transported to the laboratory in a few days. Once back to the laboratory, the filters and sediments were frozen at − 80 °C until DNA extraction.

Additionally, at each site, triplicate sediment samples were collected in sterile plastic bags, and triplicate water samples were collected at each site in sterile 500 mL polythene bottles. The water and sediment samples were then transported to the lab for property analyses (stored at 4 °C in dark). At the time of sampling, water temperature (T), pH, dissolved oxygen (DO), conductivity (COND), turbidity (TURB), total dissolved solids (TDS), and oxidation-reduction potential (ORP) were measured on-site using HORIBA-U52 multiparameters water analysis instrument (HORIBA Corporation, Japan).

### Sample physicochemical and microbial activity analysis

The total nitrogen (TN) in water samples was measured by alkaline potassium persulfate oxidation method (Ebina et al. 1983). The total phosphorus (TP) in water samples was determined by an ascorbic acid method after persulfate digestion (Zhao et al. 2015). For the sediment sample, 5 g of wet sediment samples were oven-dried at 105 °C until a constant weight was obtained (approximately 4 h) (Bratbak and Dundas 1984), and weighed (dry weight, DW) after cooled to room temperature in a desiccator. The sample was then combusted at 550 °C for 5 h in a muffle furnace. The organic matter content (OM) was calculated by subtracting the sediment weight after combustion from the oven-dry sediment weight as suggested by Heiri et al. (2001).

Simultaneous digestion method using potassium peroxodisulfate as an oxidizing reagent was used to determine the concentration of TN and TP in sediment samples (Luo et al. 2019). Briefly, 100 μL 95% ethanol was transferred to wet approximately 150 mg grinded sediment sample in an oven-dried (103 °C, 3 h) corundum crucible. The corundum crucible with sediment sample was heated at 720 °C for 15 min after the addition of 2 g NaOH. The above sample was cooled, 100 mL of water added, and filtered (0.45 μm). TP (mg g dry weight (DW)^−1^) and TN (mg gDW^−1^) concentrations in above filtered water were then determined by spectrophotometer (UV-5500, Metash Corporation, Shanghai, China) after digestion with alkaline potassium persulfate solution (Ebina et al. 1983).

Sediment microbial activity (MA) was analyzed by the hydrolysis of fluorescein diacetate (FDA) spectrophotometric method (Schnürer and Rosswall 1982). Briefly, FDA was dissolved in 50 mL of acetone, and stored at − 20 °C. One gram sediment sample was dissolved in approximately 10 mL sterilized potassium phosphate buffer pH = 7.4 in a 150-mL flask, incubated at 30 °C for 3 h in dark on a rotary shaker after addition of 0.5 mL FDA stock. Another 25 mL of acetone (50% vol/vol) was added to the 150-mLflask to terminate the reaction. The flask was stoppered and the contents shaken vigorously for 30 s by hand. The mixture was then filtered (0.45 μm) and the filtrates measured at 490 nm on a spectrophotometer (UV-5500, Metash Corporation, Shanghai, China). The total microbial activity (μg FDA g^−1^ sample DW h^−1^) was calculated using the calibration curve produced from standards.

### DNA extraction and Illumina Hiseq2500 sequencing of 16S rRNA gene amplicons

DNA was extracted from 0.2 g of fresh sediment sample using an E.Z.N.A soil DNA kit (Omega Biorek Norcross, GA, USA). For water samples, the 0.2 μm micropore membranes with the water sample filter residues were cut into small pieces. Then, water DNA was extracted from the small pieces using an E.Z.N.A soil DNA kit (Omega Biorek Norcross, GA, USA). Finally, the DNA extracts were diluted to 10 ng μL^−1^ for subsequent operations.

The PCR amplification of the 16S rRNA gene for the Illumina Hiseq sequencing was performed using an ETC-811 PCR amplifier (Eastwin, Beijing, China) with primers 341F: CCTACGGGNGGCWGCAG and 806R: GGACTACHVGGGTATCTAAT. A barcode of 8 bp was linked to the 5′ end of the primer. The 50 μL of PCR reaction system contained 1 μL of Taq enzyme, 1 μL of template DNA, 3 μL of primer mixture, 5 μL of dNTP mixture, 5 μL of PCR reaction buffer, and 35 μL of PCR-grade water. The PCR runs started with an initial denaturation of 10 min at 95 °C, and then cycled for 30 times with 30 s of denaturation at 95 °C, 30 s of anneal at 56 °C, 40 s of extension at 72 °C, and finally extended for 10 min at 72 °C. Then, the PCR products were purified using an AMPure XP Beadskit, and then mixed with equal morality for Illumina sequencing. The Illumina Hiseq 2500 sequencing of 16S rRNA gene amplicons was performed following the standard procedure.

### Bioinformatical analysis

The 16S rRNA gene sequences were analyzed following the default UPARSE pipeline (Edgar 2013), as detailed by Che et al. (2018). Briefly, an operational taxonomic unit (OTU) table was generated at a similarity threshold of 97%. The taxonomy information of the centroid sequence of each OTU was obtained according to the latest Silva database. Then, the 16S rRNA gene sequences in each sample were rarefied to 393.82 for all the subsequent analysis. The sequences data reported in this study was archived in the Sequence Read Archive (SRA) with the accession number PRJANA636956. We calculated the richness, Chao 1, and Shannon index of water and sediment bacterial communities were performed in RStudio Desktop 1.1.463 with the vegan package (Oksanen et al. 2017). The bacterial functional profiles were predicted using Functional Annotation of Prokaryotic Taxa (FAPROTAX; version 1.1) (Louca et al. 2016).

### Statistical analysis

All the environmental variables, with an exception of pH, were log_10_(*x*+1) transformed to improve their normality prior to the statistical analyses. The chemical data of the water and sediment were compared using one-way ANOVA or repeated measures ANOVA followed by Tukey’s post hoc test for multiple comparisons. Pearson’s correlation analysis was used to test relationships among environmental variables and bacterial richness of water and sediment samples in two seasons. The one-way ANOVA, repeated measures ANOVA, and Pearson’s correlation analysis were conducted using SPSS (version 20, SPSS Inc., Chicago, USA).

The comparison of microbial communities at different sampling sites was assessed using permutational multivariate analysis of variance (PERMANOVA). Non-metric multidimensional scaling (NMDS) was performed to display the dissimilarity of OTU-level community composition of water and sediment bacteria among the samples. Mantel test was conducted to examine the relative impacts of environmental variables on community composition. The PERMANOVA, NMDS, and Mantel test were conducted using the vegan package in R software (Version 1.1.463, Boston, Massachusetts, USA). Relationship between environmental variables and bacterial community structure was also analyzed using forward selection, then a parsimonious redundancy analysis (RDA) was conducted with those selected variables in Canoco software (Version 5) (ter Braak and Smilauer 2012).

## Results

### Physical and chemical characteristics of water and sediment samples

The physicochemical characteristics of surface water and sediment samples are shown in Table S2. Water chemistry showed no significant difference between S1 and upstream and downstream samples of S2 with the exception of T. Physical-chemical properties of the water column did not display clear distinctions between upstream and downstream locations from dams, for example, S2-U vs S2-D1, S3-U vs S3-D, and S6-U vs S6-D. At S2, differences in physicochemical characteristics of both water and sediment from upstream to downstream of S2 was significant, with the exception of T and pH for water samples (Table 1). Additionally, significant differences in physicochemical properties of both water and sediment were observed between winter and summer, with the exception of OM for sediment samples (Table 1).

**Table 1:** *p* values from repeated measures ANOVA for the factors season as a repeated factor and the sampling site (Site) as a fixed factor during the experimental period (winter and summer)

Water												
Source of variation		T	pH	DO	ORP	COND	TURB	TDS	TN	TP	N:P	Richness
Site	*p*	0.298	0.404	< *0.001*	*0.003*	< *0.001*	< *0.001*	< *0.001*	0.174	< *0.001*	*< 0.001*	*0.014*
	F	1.37	1.05	129.27	42.82	79.09	1263.99	183.16	2.27	21.34	43.31	11.48
Season	*p*	< *0.001*	*0.034*	< *0.001*	< *0.001*	< *0.001*	< *0.001*	< *0.001*	< *0.001*	*0.003*	< *0.001*	< *0.001*
	F	152.78	10.04	2486.60	1232.52	5750.36	2382.89	8320.01	273.06	42.25	145.80	2042.70
Site × Season	*p*	0.983	0.717	< *0.001*	*0.014*	<*0.001*	< *0.001*	< *0.001*	0.428	0.115	0.114	*0.006*
	F	0.05	0.46	54.37	17.75	41.33	1042.54	110.84	0.93	2.43	2.45	17.71
Sediment												
Source of variation		WC	OM	MA	TN	TP	N:P	Richness				
Site	*p*	< *0.001*	*0.006*	*0.023*	*0.027*	< *0.001*	*0.003*	*0.006*				
	F	134.76	6.77	12.34	4.35	39.34	8.48	14.46				
Season	*p*	*0.003*	0.419	< *0.001*	*0.044*	< *0.001*	*0.032*	*0.039*				
	F	39.95	0.81	1122.95	3.65	1257.96	10.52	9.16				
Site × Season	*p*	0.058	0.101	*0.020*	*0.040*	*0.007*	*0.022*	0.083				
	F	3.29	2.59	13.24	8.98	6.53	4.64	3.94				

TP and N:P ratio were negatively correlated in winter and summer seasons (*r* = − 0.6, *p* < 0.05) using Pearson’s test (see Supplementary Fig. S1), suggesting that TP is the limiting factor in water. DO, COND, and pH of water samples fluctuated and decreased from S1 to S8, and pH and COND peaked at S1 in winter. In sediment samples, TN and TP exhibited similar trends, and were closely correlated and generally increased along the river during both winter (*p* < 0.01 for TP, *p* = 0.035 for TN) and summer (*p* = 0.01 for TP, *p* < 0.01 for TN). Moreover, TN and TP contents of sediment samples in winter were much lower than during the summer.

### Variation of *α* diversity and microbial community composition

**Fig. 2:**
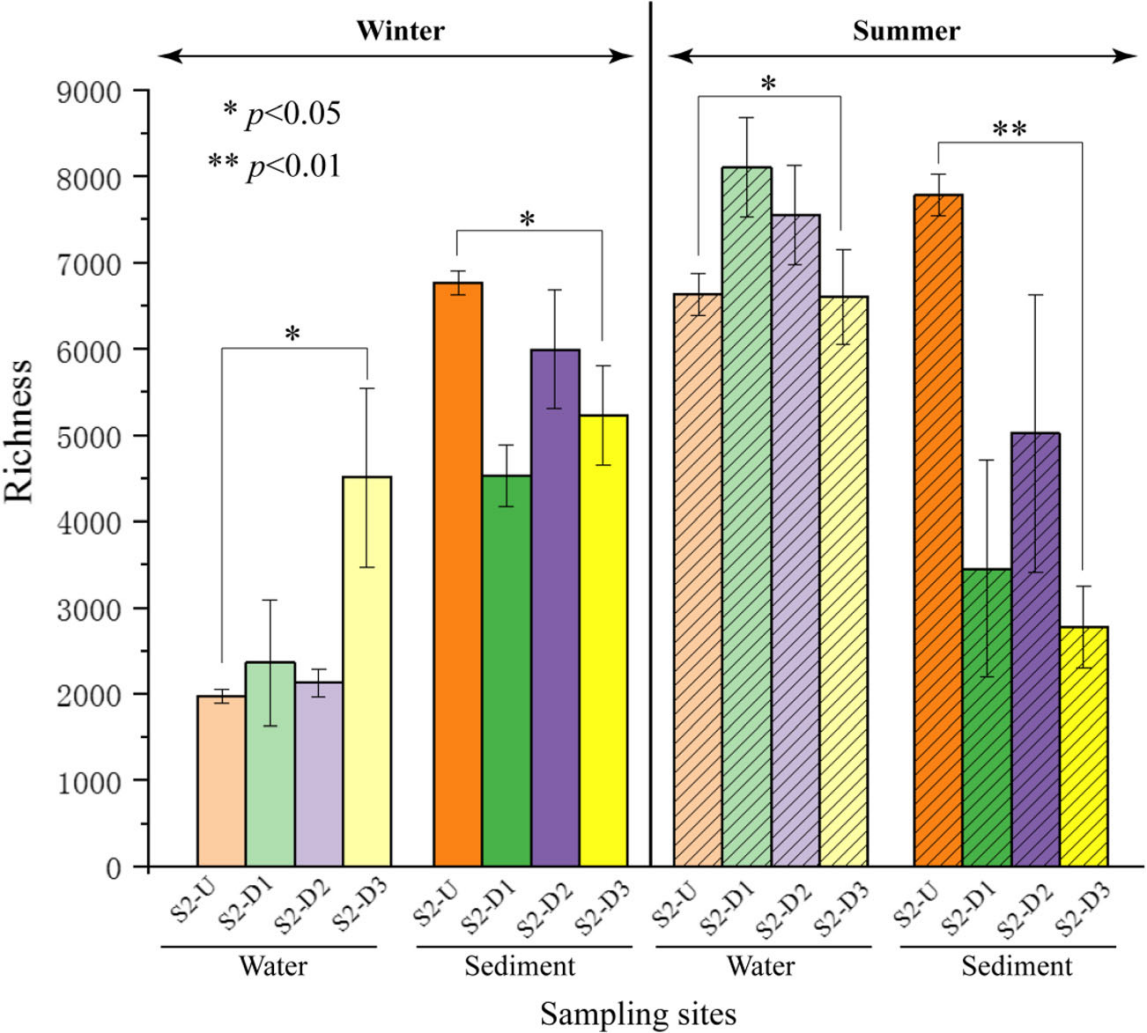
Bacterial richness in water and sediments from upstream to downstream of S2 at two seasons

**Fig. 3:**
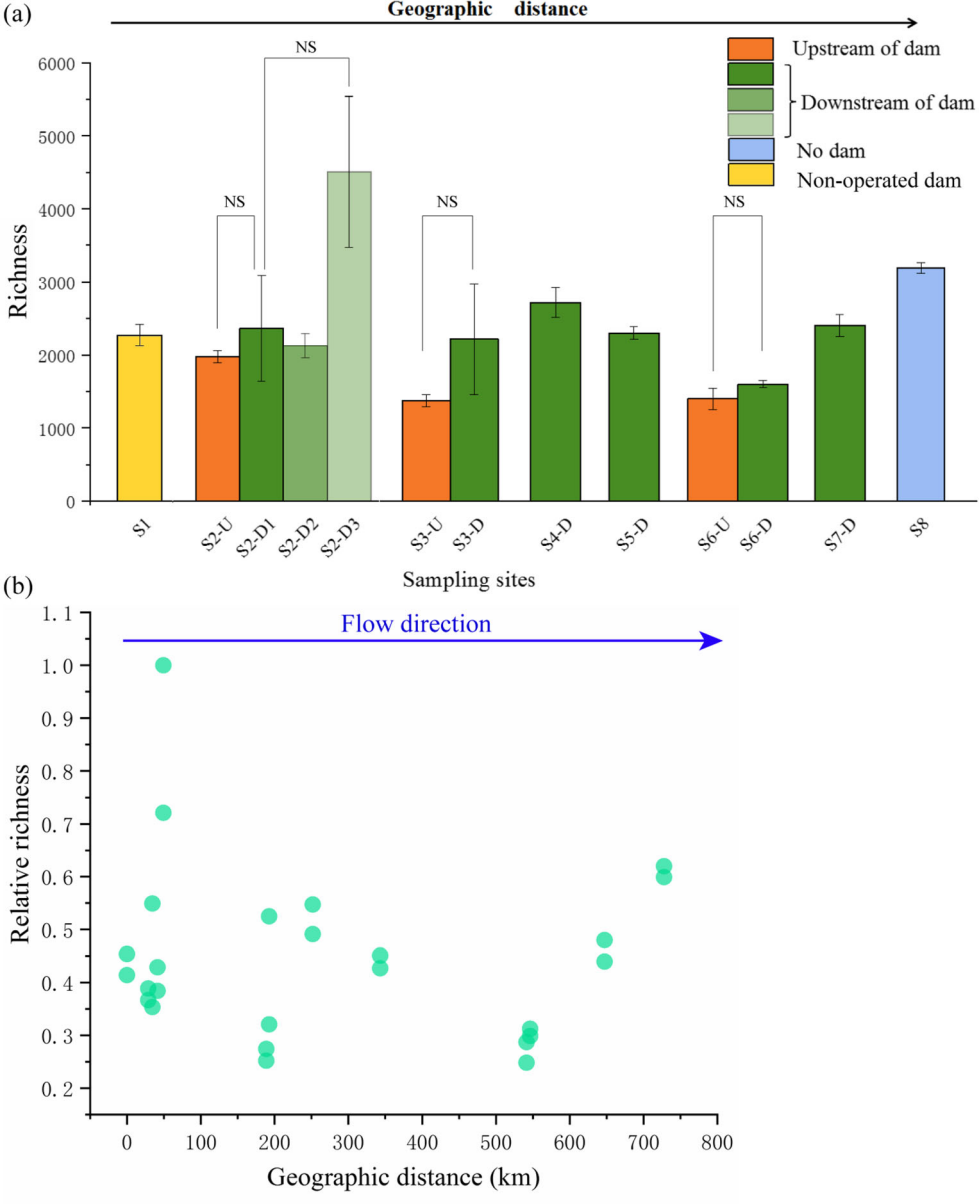
Bacterial richness (**a**) and relative richness (**b**) in water from the total 13 sampling sites along the Lancang River from upstream to downstream. Relative richness is the ratio between the richness at this site and the maximal richness of all sites. Error bar indicates one standard deviation of two replicates samples at each sampling site

A total of 4,966,533 sequences were generated by the high throughput sequencing. Sequences per sample ranged from 40,431 to 182,101. Filtered sequences were clustered into 42,737 OTUs at 97% sequence similarity. Based on current sequencing depth, most biodiversity was recovered according to the rarefaction analysis (Supplementary Fig. S2). Richness significantly changed from upstream to the downstream of S2 in winter and summer for both sediment and water samples (Fig. 2 and Table 1). However, bacterial richness did not differ significantly between sampling points upstream (e.g., S2-U) and immediately downstream (e.g., S2-D1) of the dam as shown in Fig. 3. Four indices of *α* diversity, including Shannon, Chao 1, ACE, and Pielou’s evenness, were calculated to quantify both species richness and evenness (Table 2). *α* diversity of samples from the water column increased slightly from upstream toward downstream along the river, but this trend was not statistically significant. In contrast, *α* diversity of sediment samples decreased from upstream toward downstream of S2 in both winter and summer. Shannon index (*p* = 0.03), Chao 1 (*p* < 0.01), and ACE (*p* < 0.01) indices decreased significantly in summer, and Chao 1 (*p* = 0.025) and ACE (*p* = 0.037) decreased significantly in winter. Moreover, sediment samples had significantly higher *α* diversity than water samples in winter (*p* < 0.05). This situation was reversed in summer, where water samples displayed higher *α* diversity (*p* < 0.05) than corresponding sediments. No significant seasonal difference was found in sediment samples in terms of *α* diversity, but *α* diversity of aquatic bacterial communities was significantly higher in summer than winter.

**Table 2:** Summary of high throughput sequencing of 16S rRNA genes in water and sediment samples

	Season	Sample ID	OTUs number	Shannon	Chao1	ACE	Pielou’s evenness
Water	Winter	S1	2272	4.39	4198	4476	0.57
		S2-U	1983	4.22	4318	4539	0.56
		S2-D1	2349	4.26	4858	5258	0.55
		S2-D2	2115	4.29	3815	4211	0.56
		S2-D3	4528	5.62	8442	8958	0.67
		S3-U	2225	5.05	3852	3949	0.66
		S3-D	1375	5.23	2311	2345	0.72
		S4-D	2708	5.90	4195	4284	0.75
		S5-D	2293	5.56	4199	4286	0.72
		S6-U	1602	4.92	2450	2404	0.68
		S6-D	1422	5.37	2704	2695	0.73
		S7-D	2401	5.42	3972	4048	0.70
		S8	3232	5.76	5099	5350	0.71
	Summer	S2-U	6598	6.42	11195	11908	0.73
		S2-D1	8181	7.04	13741	14577	0.78
		S2-D2	7620	6.70	13001	13873	0.75
		S2-D3	6661	7.03	9890	10061	0.80
Sediment	Winter	S2-U	6717	7.45	9871	10089	0.84
		S2-D1	4545	6.44	6224	6132	0.77
		S2-D2	5966	7.04	8898	9170	0.81
		S2-D3	5210	6.31	8318	8450	0.74
	Summer	S2-U	7805	7.77	11704	11995	0.87
		S2-D1	3496	5.66	5394	5137	0.70
		S2-D2	5039	6.76	7502	7333	0.80
		S2-D3	2769	6.25	3903	3810	0.79

**Fig. 4:**
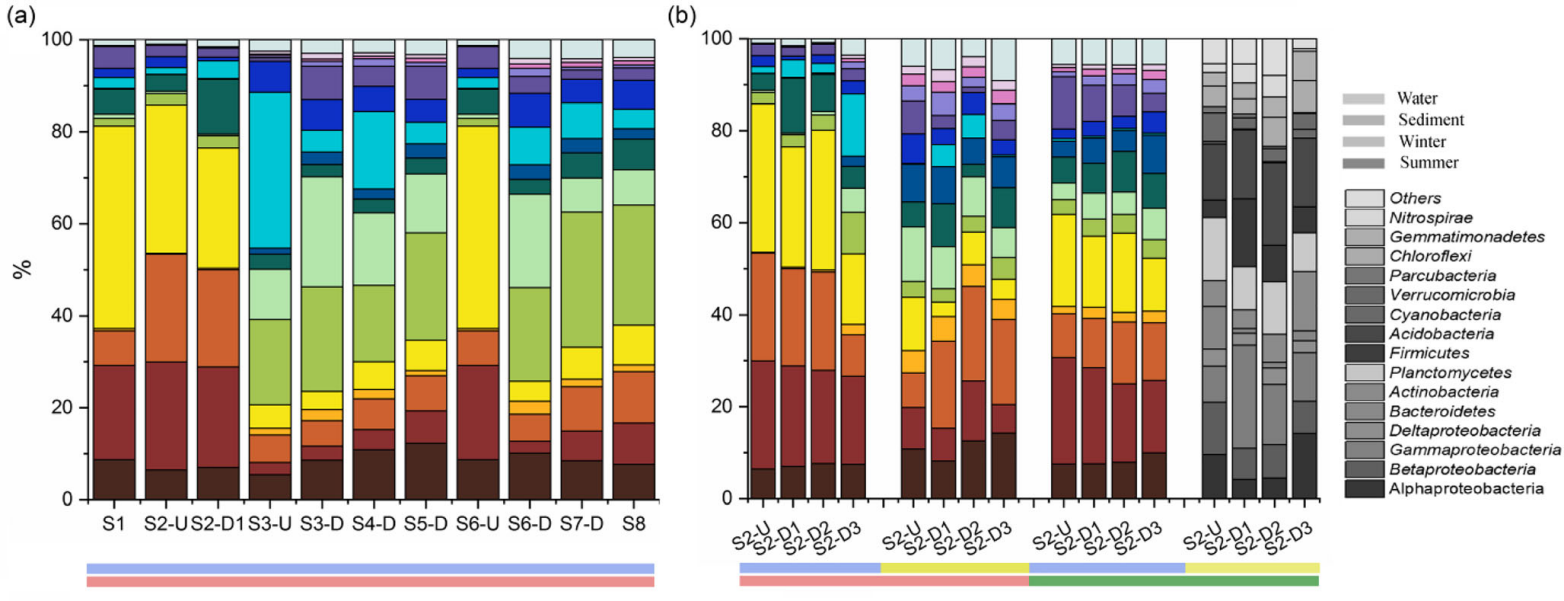
Community composition at the phylum taxonomic level for all 22 water samples from 11 sites (*n* = 2 for each bar) along the Lancang River collected during winter (**a**), and 8 water samples, 8 sediment samples from 4 sites (*n* = 2 for each bar) along the Gongguoqiao hydroelectric dam collected during winter (**b**, ), 12 water samples, 12 sediment samples from 4 sites (*n* = 3 for each bar) along the Gongguoqiao hydroelectric dam collected during summer (**b**, )

Bacterial community composition varied at phylum taxonomic levels as shown in Fig. 4. Dominant phyla in flowing and ponded sites, including the sediment and water samples, were *Proteobacteria* (mostly classes of *Alphaproteobacteria*, *Betaproteobacteria*, and *Gammaproteobacteria*) (35.8%), *Bacteroidetes* (12.4%), *Actinobacteria* (9.6%), *Planctomycetes* (8.5%), and *Firmicutes* (6.2%) accounting for 72.5% of bacterial sequences (Fig. 4a). A relatively higher abundance of *Betaproteobacteria*, *Gammaproteobacteria*, and *Bacteroidete* was detected in the water samples from S1, S2, and S6-U (Fig. 4b). The bacterial composition of water samples collected during the summer displayed a significantly increased abundance of *Deltaproteobacteria* and *Planctomycetes* compared with winter samples. In contrast, bacterial communities in sediment samples from upstream and downstream of S2 during winter and summer were quite similar; only *Acidobacteria* showed significantly higher abundance during summer (*p* < 0.001).

### Spatial variation of bacterial community and the correlation with environmental variables

**Fig. 5:**
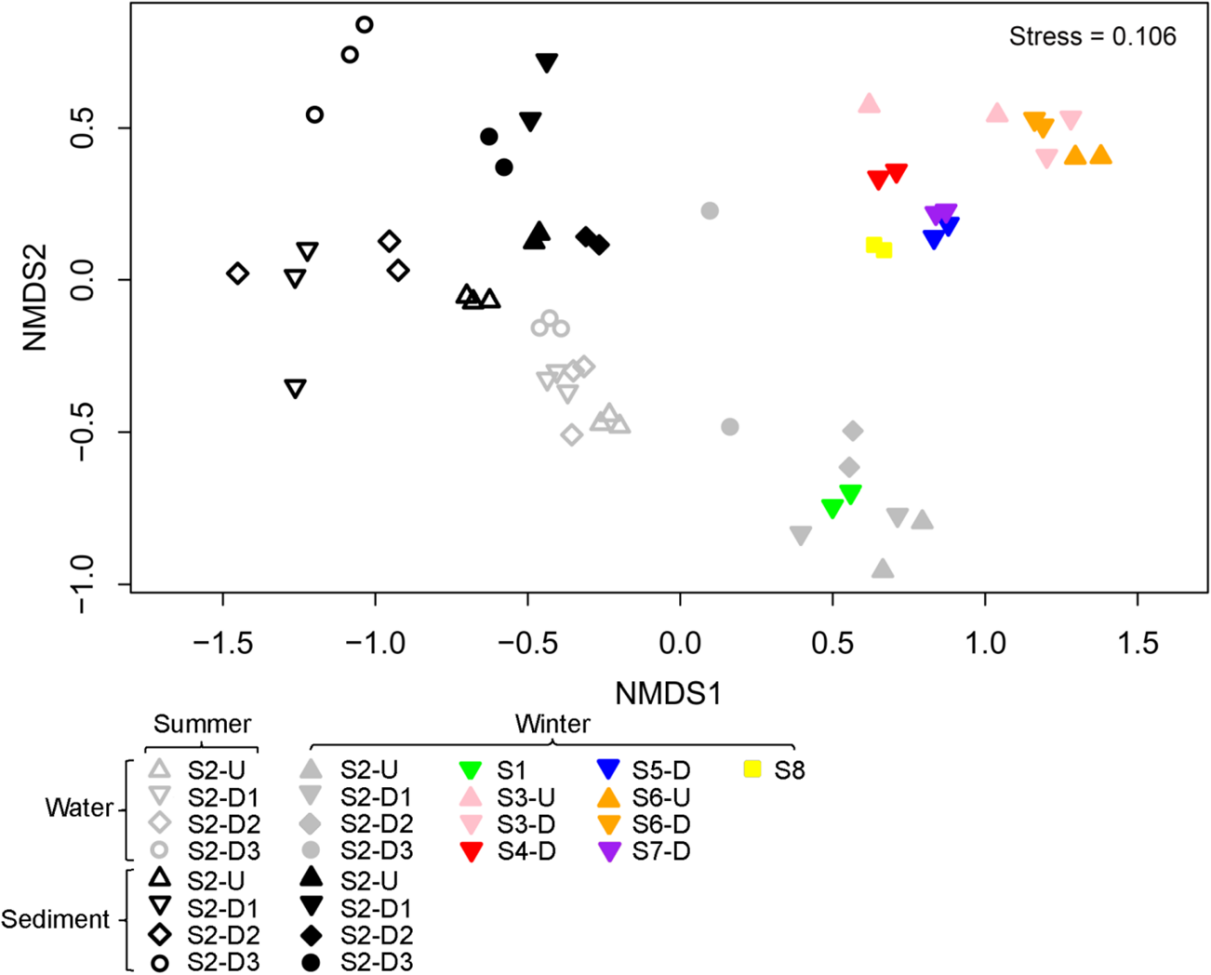
Bacterial community composition of all water and sediment samples as indicated by NMDS plot

NMDS plots were applied to investigate the spatial variation at 13 sampling sites, based on Bray-Curtis distances according to OTU distribution (Fig. 5). Findings revealed complex variability of bacterial communities along the river and in different habitats during different seasons. Clusters were less distinct for water samples collected from upstream and downstream of the hydropower station. Water samples from S1 and S2 were grouped and separated from all remaining sites (i.e., S3–S8) in winter (Fig. 5). Mantel tests suggested a positive correlation between geographic distance and bacterial community structure across all samples (*r* = 0.4826, *p* = 0.001). Temporal changes in winter samples were observed for water samples collected from S2 (Fig. 5). Moreover, summer samples showed highly similar bacterial community composition, regardless of samples collected upstream and downstream of the hydropower station. However, between-site differences were highly significant for winter samples (Fig. 5). Similarly, findings from sediment samples collected from different seasons were distinctly different. By focusing dam effects, we found sediment samples from upstream to downstream of the hydropower station were divergent between in winter and summer. In addition, results from sediment samples were clearly distinct from water samples in winter and summer seasons. Analysis by PERMANOVA confirmed that water microbial communities did not differ significantly between upstream and downstream sites (i.e., S2-U vs S2-D1, S3-U vs S3-D, S6-U vs S6-D) in winter (*r*^2^ = 0.074, *p* = 0.567). Furthermore, water from upstream (S2-U) and downstream (S2-D1) of S2 did not have significantly different bacterial community profiles in summer (*r*^2^ = 0.26, *p* = 0.1), while sediment bacterial communities differed significantly between upstream and downstream sites in summer season (*r*^2^ = 0.22, *p* = 0.01).

**Fig. 6:**
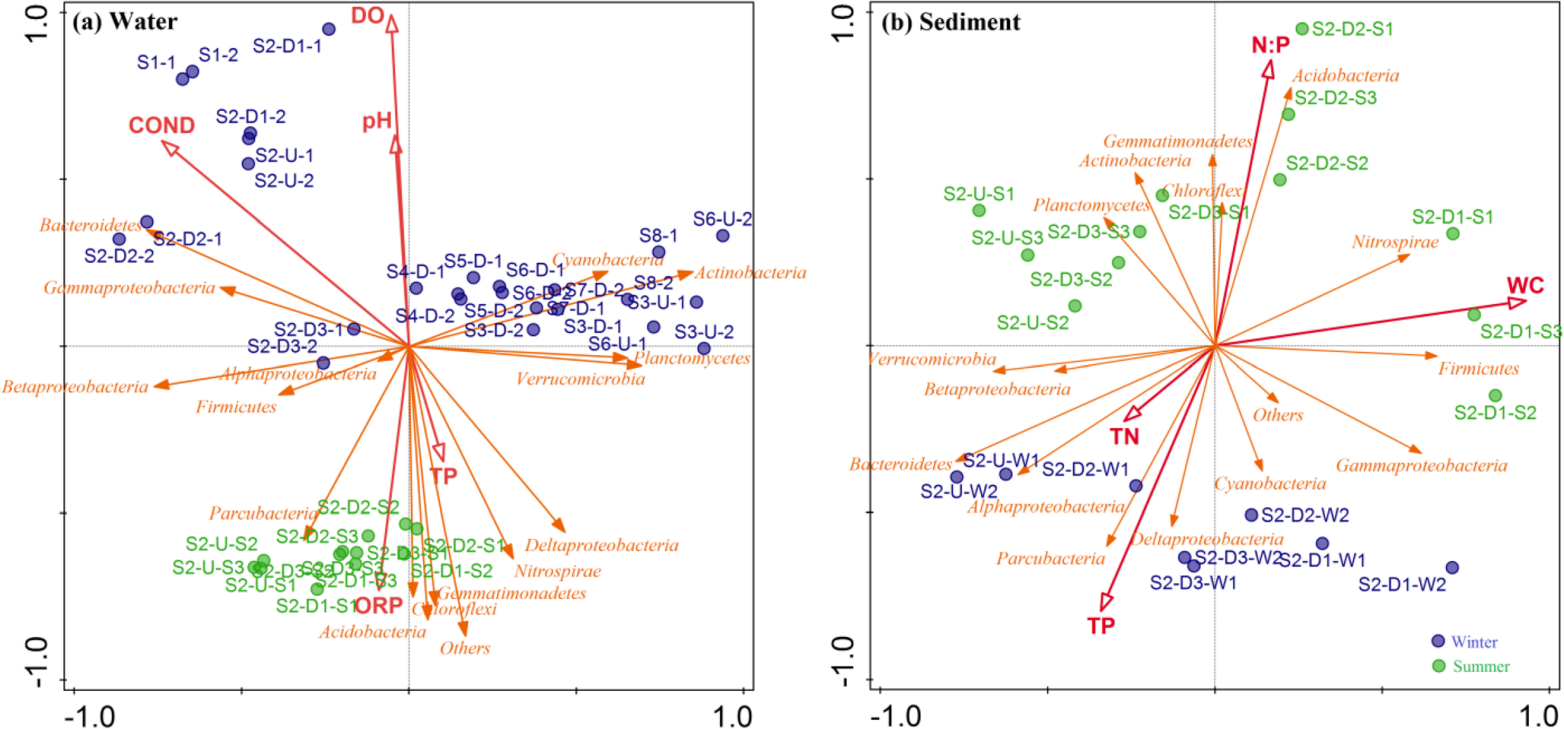
RDA of the bacterial community and the most significant physiochemical variables shaping bacterial community composition and structure in water (**a**) and sediment (**b**) along the Lancang River. Symbols indicated sampling sites. Arrows indicate environmental variables and bacterial community composition in phylum level. The lengths of the arrows indicated how much variance was explained by the corresponding variable. The angles between arrows indicate correlations between individual environmental variables

Pearson analysis indicated that the bacterial richness in the water column along the river was negatively correlated with N:P, TN, and DO, and positively correlated with ORP (Supplementary material Fig. S1). Bacterial richness of sediment samples was strongly correlated with WC. However, richness was not linked to geographic distance (Fig. 3). A Mantel test identified environmental factors more highly correlated with bacterial community structure in water samples (*p* < 0.05; Table 3). The RDA analysis (Fig. 6) showed COND had the most pronounced impacts on bacterial community composition in water samples, while WC was most prominent for sediment samples. Among five selected chemical variables, major contributor variation in bacterial populations included COND, DO, and ORP. For sediment samples, WC, TN, TP, and N:P were important for distinguishing findings from winter and summer samples (Fig. 6b).

**Table 3:** The correlation (*r*) and significance (*p*) were determined by Mantel tests between physiochemical variables and microbial community composition in water and sediments in winter and summer

Variable	Water					
	Winter^1^		Summer^2^		Total^3^	
	*r*	*p*	*r*	*p*	r**	*p*
T	0.74**	0.001	− 0.087	0.71	0.25	0.001
pH	0.18	0.013	0.19	0.063	0.14	0.006
ORP	0.10	0.064	0.36**	0.009	0.15	0.002
COND	0.78**	0.001	0.33**	0.009	0.35	0.001
TURB	0.40**	0.001	0.3	0.025	0.35	0.001
DO	0.60**	0.001	0.085	0.27	0.46	0.001
TDS	0.80**	0.001	0.27	0.036	0.37	0.001
TN	0.15	0.029	− 0.1	0.775	0.24	0.001
TP	0.21	0.015	0.35	0.017	0.21	0.001
N:P	0.21	0.015	0.35	0.016	0.21	0.001
Variable	Sediment^2^					
	Winter		Summer		Total^3^	
	*r*	*p*	*r*	*p*	*r*	*p*
WC	0.071	0.34	0.56**	0.001	0.27**	0.006
OM	− 0.02	0.51	0.41**	0.002	0.11	0.154
TN	0.14	0.21	− 0.094	0.76	− 0.017	0.55
TP	0.031	0.42	0.36	0.01	0.28**	0.004
N:P	0.031	0.44	0.36**	0.005	0.28**	0.004
MA	− 0.17	0.73	0.014	0.49	0.12	0.12

^1^Data were from upstream and downstream of all sampling sites (i.e., S1–S8)

^2^Data were from upstream and downstream of S2

^3^It includes all data from winter and summer

**Significant correlations at *p* < 0.01

### Significant changes in functional groups along the Lancang River

**Fig. 7:**
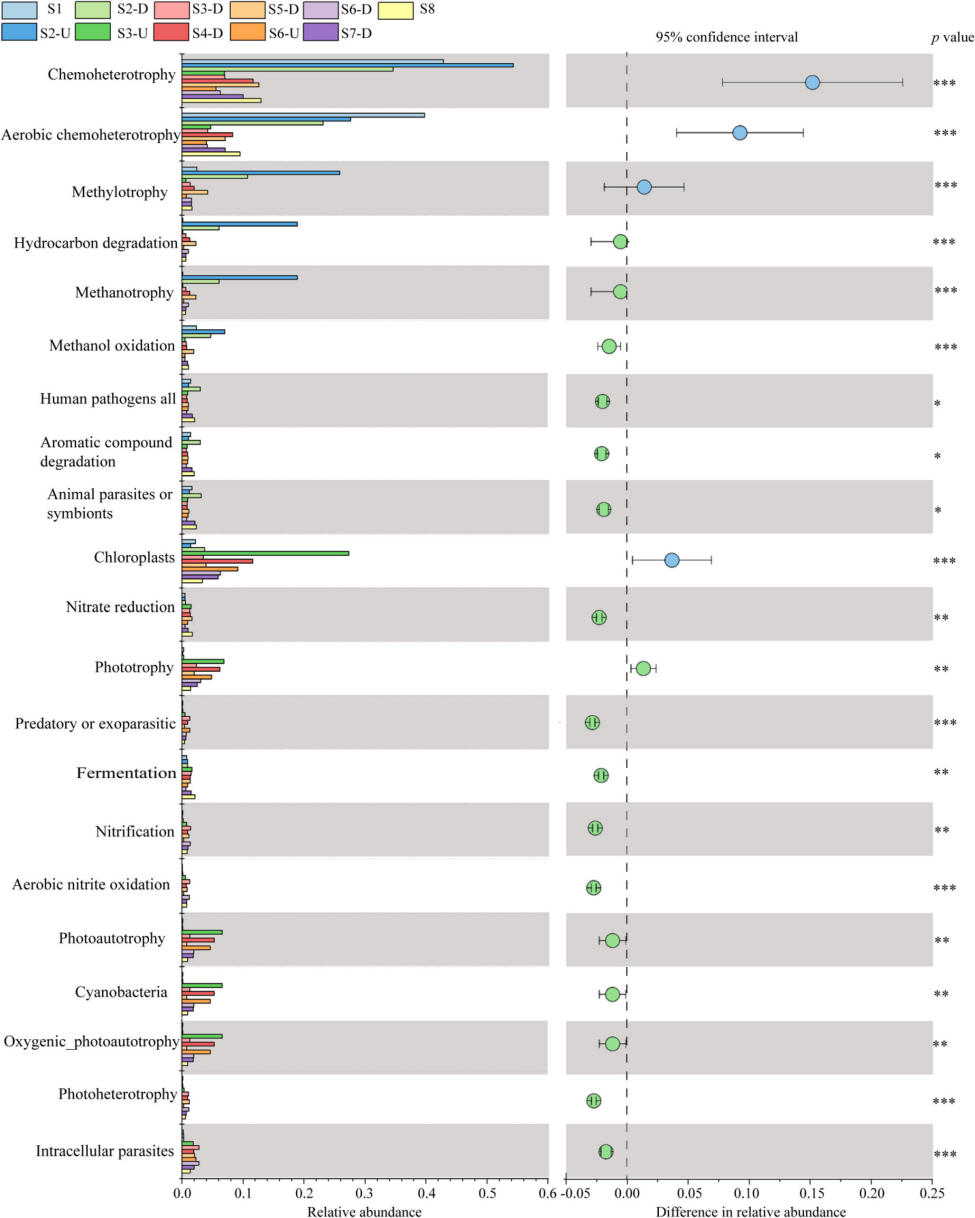
Clustered bar plots of relative OTU functional group relative abundances along the Lancang River based on FAPROTAX. The left panel displayed the abundance ratio of different functional groups; the middle showed the percentage of functional group abundance within the 95% confidence interval; the right indicated *p* value. *p < 0.05, **p < 0.01, and ***p < 0.001

A total of functional 91 groups were identified by using FAPROTAX. Dominant groups along the river in winter were associated with chemoheterotrophy, aerobic chemoheterotrophy, methylotrophy, and chloroplates; only 22 functional categories showed significantly different relative abundance (ANOVA, *p* < 0.05, Fig. 7). For samples collected from upstream and downstream of S2 in winter, dominant functional groups in both water and sediment samples were associated with chemoheterotrophy, aerobic chemoheterotrophy, methylotrophy, hydrocarbon degradation, and methanotrophy (Supplementary Fig. S3a, b). Moreover, the relative abundance of most nitrogen metabolic OTUs (e.g., nitrification, nitrate reduction, nitrite oxidation, nitrate/nitrogen respiration) and carbon metabolic OTUs (e.g., methanotrophy, hydrocarbon degradation, methylotrophy) were altered significantly in water samples between seasons. Most functional groups associated with C cycling in sediment samples decreased from winter to summer (*p* < 0.05). N:P ratio in water showed the highest correlations with most nitrogen cycle groups.

## Discussion

### Spatial distribution of microbial communities in surface water influenced by cascade dams along the Lancang River

We investigated spatial distributions of bacterial communities in surface water over a 720 km reach of the river. Our observations on the wide spatial distribution of bacterial taxa in surface water along the Lancang River indicated that bacterial community structure was largely dependent on geographic distance, as shown by the Mantel test. This finding contrasts with patterns reported by Fierer et al. (2007), who indicated that dispersal limitations have little influence on biogeographical distributions of stream bacteria within the Hubbard Brook watershed (total channel length of approximately 270 km). Differences could be due to the presence of cascade dams and a larger spatial scale in our current study. Dams are widely acknowledged for fragmenting rivers, resulting in disruption of natural bacterial dispersal (Algarte et al. 2016; Jansson et al. 2000). Source populations of microorganisms and habitats are prone to isolation by cascade dams, thus limiting dispersal. Moreover, neutral processes and dispersal limitations seem to be generators of *β* diversity at larger spatial scale (Declerck et al. 2011; Heino et al. 2015; Van et al. 2007). These observations may reflect that spatial scale is associated with between-habitat distances, hydrological connectivity, and physical structure of landscapes. Such factors are vital in determining types and strengths of alternative spatial dynamics that shape metacommunity structure (Dumbrell et al. 2008).

Aquatic bacterial community structure between sampling sites upstream (i.e., dam reservoir) and immediately downstream of a single hydroelectric dam showed great similarity in our study. This result might be explained by connectivity between the reservoir and downstream sites. Dams, especially daily operated dams like Gongguoqiao, Dachaoshan, Manwan, and Jinghong along the Lancang River, may also increase flow similarity and thus impose environmental homogeneity over natural flow regimes (Poff et al. 2007). Our study also shows that bacterioplankton diversity is unrelated to geographic location along the river corridor. Inflow from tributaries in the watershed could explain increasing microbial diversity along the river. Furthermore, a shift of dominant phyla in water was observed at S1, S2, and S6 in winter. Bacterial community composition at S1, S2, and S6-U in winter was dominated by the phyla *Proteobacteria*, *Bacteroidetes*, and *Firmicutes.* In contrast, microbial communities at the remaining sites were dominated by the phyla *Proteobacteria*, *Actinobacteria*, *Planctomycetes*, and *Cyanobacteria*. Hydroelectric dams examined in this study differed with regard to the age of construction. S2 and S6 were constructed in 2013 and 2014, respectively, while the dam reservoir at S1 had not begun filling at the time of sampling. Higher amounts of readily degradable organic matter and nutrients are expected in newly constructed reservoirs than in older ponds (St. Louis et al. 2000). High organic matter in sediments may create new conditions that favor non-native and otherwise poorly adapted species, and thus differentially contribute to observed levels of dominant phyla at different sampling locations.

We find evidence that turbidity is not only positively linked to bacterial richness (Fig. S1), and is also an important explanatory variable for community structure (Table 3) along the river in winter. One hypothesis is that suspended particles may adsorb some nutrients in turbid water, and communities on particles may support some bacteria that are not present or in low abundance in free-living communities (Wang et al. 2012a). Changes in most physicochemical properties between reservoir and riverine sites were evident, but dominant bacterial community structure at the phylum level in each reservoir was not different from that at the riverine stations, except the site S6. As the largest dam on the Lancang River, Nuozhadu dam at S6 is characterized as a yearly operated dam and experiences greatest changes in seasonal streamflow, and dominant bacterial assemblies downstream of S6 showed dramatic differences compared with bacterial communities in the reservoir.

Additionally, the influence of hydroelectric dams is likely to facilitate water eutrophication, and thus possibly promote *Cyanobacteria* blooms (Wang et al. 2018) (Fig. 4). Significantly higher *Actinobacteria* and *Planctomycetes* were associated with organic carbon deposition (Cao et al. 2017; Kolehmainen et al. 2008), indicating that damming might promote utilization of refractory organic matter (Haukka et al. 2005). Predominant phyla in water in winter at S1, S2, and S6-U, *Bacteroidetes* and *Firmicutes* are also dominant in mammalian (Duncan et al. 2008; Ley et al. 2008) and human (Duncan et al. 2008) gut. Some species from these phyla may be fecal indicators (Jeong et al. 2011). The significant high abundance of these phyla could reflect untreated wastewater from agricultural runoff, residential, and wildlife areas. We also note significantly lower microbial diversity in sediments immediately downstream of a hydroelectric dam. A possible reason is the increased habitat heterogeneity in sediment habitats in dam reservoirs (Prchalová et al. 2009) that may induce significant spatial differences in bacterial communities between upstream and downstream locations. Similar trends in bacterial community composition in surface water and sediments were also found in the Yangtze River (Liu et al. 2018).

Functional groups taxa associated with carbon metabolic OTUs, including methylotrophy, hydrocarbon degradation, methanotrophy, and methanol oxidation, constituted a substantial fraction of functionally annotated OTUs in the upper reaches of the Lancang River (Fig. 7). In contrast, more genes associated with photoautotrophs were abundant gene groups in the lower reaches of the river. Thus, autotrophs are important members of microbial communities farther downstream.

### Seasonal variability in the composition and abundance of microbial communities

Variations in aquatic and sediment communities during winter and summer seasons were determined by Illumina Miseq sequencing from upstream to downstream of the Gongguoqiao hydroelectric dam (i.e., S2). Overall species richness and diversity were lower in winter than in summer consistent with previous studies (Feng et al. 2016). Significantly higher richness in bacterial communities in sediments in the reservoir of the Gongguoqiao dam was observed as expected. However, richness in bacterial communities in surface water did not differ significantly at sampling sites upstream and immediately downstream of the dam. The OTU richness in sediments was significantly lower than in corresponding water samples in summer, presumably due to the increased loading of microorganisms bound to particulate matter following nonpoint source runoff and stream bottom resuspension (Atherholt et al. 1998).

The effect of season on shaping community structure in water and sediment was analyzed by NMDS diagramming and microbiome profile analysis. The results indicate a temporal community structure, with more homogeneity in the summer samples than in winter samples. De Oliveira and Margis (2015) suggested that the increased rainfall, with a concomitant increase in river flow and homogenation, could explain this temporal pattern. In terms of microbiome profile, the planktonic microbial community in both winter and summer seasons was characterized by a high proportion of *Proteobacteria* and *Bacteroidetes*. However, unlike the sedimentary communities, in which only the *Acidobacteria* were more enriched in summer, the main phyla in water varied according to sampling seasons. For example, the abundances of *Deltaproteobacteria*, *Planctomycetes*, and *Acidobacteria* in summer were higher than in winter, whereas *Cyanobacteria* were more prevalent in winter. *Deltaproteobacteria*, *Planctomycetes*, and *Acidobacteria* are typically found in freshwater (Wang et al. 2018) or less eutrophic environments (Liu et al. 2009). Freshwater input increased with enhanced precipitation, and abundance of these phyla was expected to increase substantially in summer. A lower TN concentration in water is an additional reason for high levels of *Planctomycetes* in summer. *Planctomycetes* may be involved in the degradation of recalcitrant organic matter (Cao et al. 2017), particularly when supplied with N in nutrient-poor waters (Tadonléké 2007). Temperature and nutrient concentrations, especially TN concentration, changed significantly from winter to summer, and these factors strongly influence the composition of bacterioplankton communities. In contrast, sediment bacterial communities exhibited little difference across seasons. Liu et al. (2018) identified a similar pattern in water and sediment in the Yangtze River. They concluded that the introduction of exotic species originating from upstream freshwater sources and terrestrial species through the various hydrologic processes occurred in different seasons and might be responsible for seasonal fluctuations in planktonic bacterial communities. Notably, spatial variability in bacterial communities across their sampling range was minimal compared with temporal differences, suggesting that seasonal changes, such as temperature and precipitation, are dominant factors in determining community composition.

Furthermore, ORP, COND, TURB, TP, and N:P were shown to be key factors associated with changes in aquatic bacterial communities in two seasons, and WC, TP, and N:P were highly correlated with variations in sediment bacterial community in these seasons. During the winter season, concentration levels of nutrients likely caused by low river flow and rare precipitation (Liu et al. 2013) contributed to changes in microbial community composition through an increasing abundance of nutrient-adapted microorganisms. The RDA analysis further indicated that pH and DO were notably associated with alterations in microbial community composition in surface water between seasons, consistent with previous findings of Jordaan and Bezuidenhout (2016).

Beyond seasonal community shifts, analyses of individual functional groups indicated that important functions for microbial communities in both water and sediment in winter were chemoheterotrophy, aerobic chemoheterotrophy, and carbon cycling related metabolisms. These functions are usually associated with bacteria that serve as decomposers, and contribute to in situ remediation and organic matter recycling in all ecosystems (Kämpfer et al. 1993; Peng et al. 2018).

## Conclusions

In conclusion, bacterial phylogenetic diversity and richness increased slightly in surface water along the Lancang River. Bacterioplankton exhibited similar community structure between dam reservoirs and sites immediately downstream of cascade dams. Spatial patterns in aquatic microbial community composition along the river were linked to geographic distance and environmental variables. Microbial population structure is distinct between seasons and habitats (i.e., water and sediment) and bacterial diversity, abundance, and richness decreased in winter. Bacterial communities in water were more sensitive to season change. The RDA indicated that DO, pH, and COND showed a significant impact on aquatic bacterial communities along the river. Moreover, temperature, precipitation, and nutrient levels were dominant drivers of seasonal microbial structure and functional changes upstream and downstream of hydroelectric dams. Bacteria in water and sediment in winter tend to function in areas of chemoheterotrophy, hydrocarbon degradation, and carbon cycling. Small proportions of sediment bacteria play significant roles in fermentation in winter. These observations provide new insights about microbial community-environmental condition relationships in a large international river under the influence of cascade dams. This information is crucial for understanding the impact of environmental variables and dam construction on bacterial community structure and function in riverine ecosystems. It may also greatly improve the understanding of water chemistry as well as effective monitoring programs in dammed rivers.

## Electronic supplementary material

ESM 1 (DOCX 1660 kb)
