# Supplementary material for: Bacterial community structure upstream and downstream of cascade dams along the Lancang River in southwestern China
Source: Environ Sci Pollut Res Int. 2020 Jul 28;27(34):42933–47. doi: 10.1007/s11356-020-10159-7 (PMC7603470; doi:10.1007/s11356-020-10159-7)
Supplement: Supplementary file 1 — (DOCX 1660 kb) [file 11356_2020_10159_MOESM1_ESM.docx]

Title: **Bacterial community structure upstream- and downstream of cascade dams along the Lancang River in southwestern China**

Xia Luo ^1,2^, Xinyi Xiang ^1,2^, Guoyi Huang ^1,2^, Xiaorui Song ^1,2^, Peijia Wang ^1,2^ , Yuanhao Yang^1,2^, Kaidao Fu ^1, 2,^* and Rongxiao Che^1, 2,^*

^1^ Institute of International Rivers and Eco-Security, Yunnan University, Kunming 650500, China

^2^ Yunnan Key Laboratory of International Rivers and Transboundary Eco-Security, Kunming 650500, China

***** Corresponding author: Chenggong University Town, Chenggong New district, Kunming, Yunnan Province, China, kdfu@ynu.edu.cn; Tel.: +86-0871-65034577; Fax: +86-0871-65034577

Chenggong University Town, Chenggong New district, Kunming, Yunnan Province, China, cherongxiao@ynu.edu.cn; Tel.: +86-0871-65034577; Fax: +86-0871-65034577

*
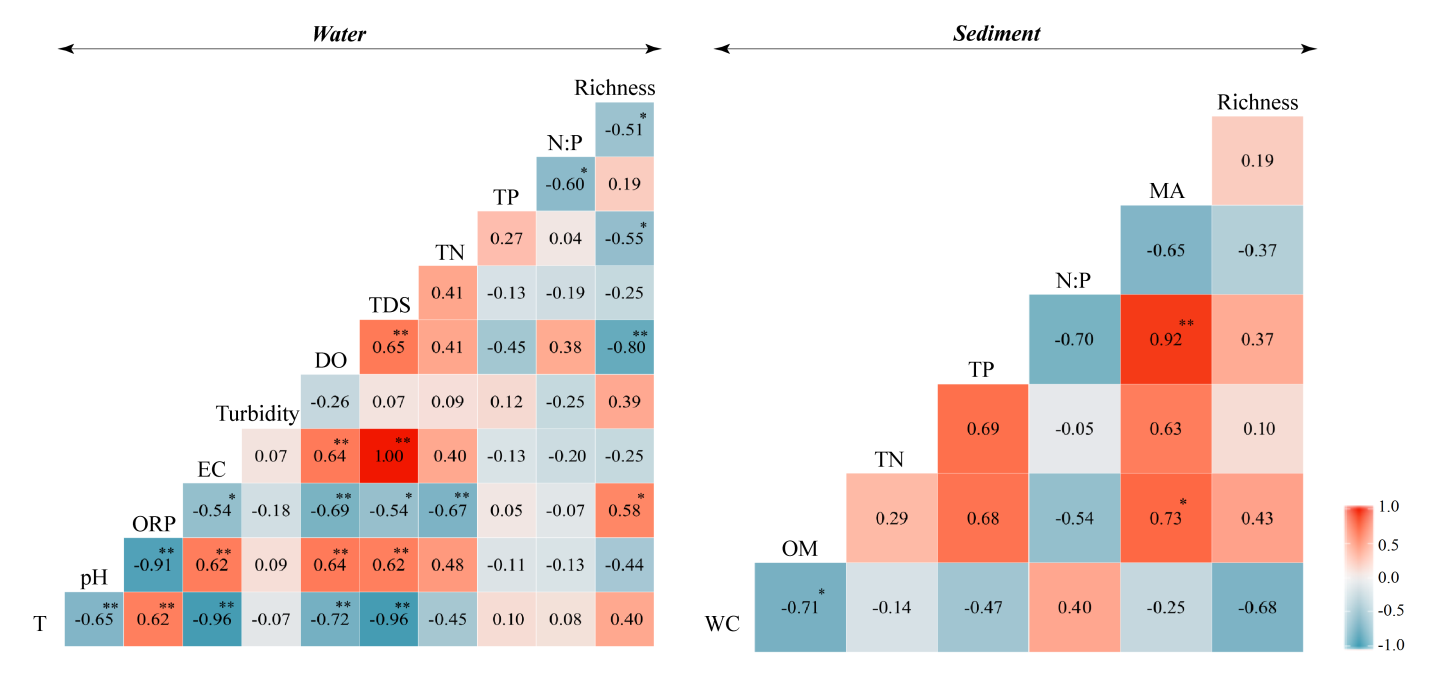
*

**Fig. S1.** Pearson correlation coefficient between bacterial richness and environmental variables of water (left) and sediment (right) in two seasons. **Significant correlations at *p* < 0.01; *Significant correlations at *p* < 0.05.


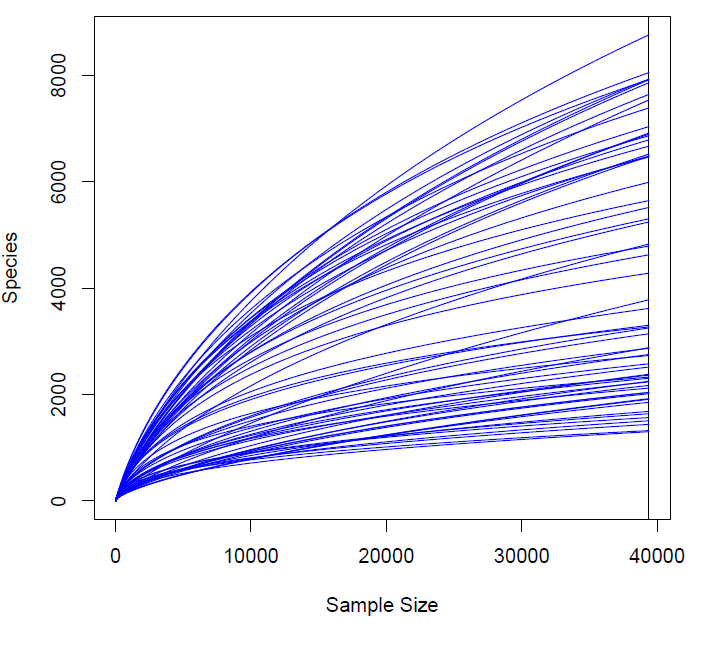


**Fig. S2.** Rarefaction curves for water and sediment samples.

**(a) Water**


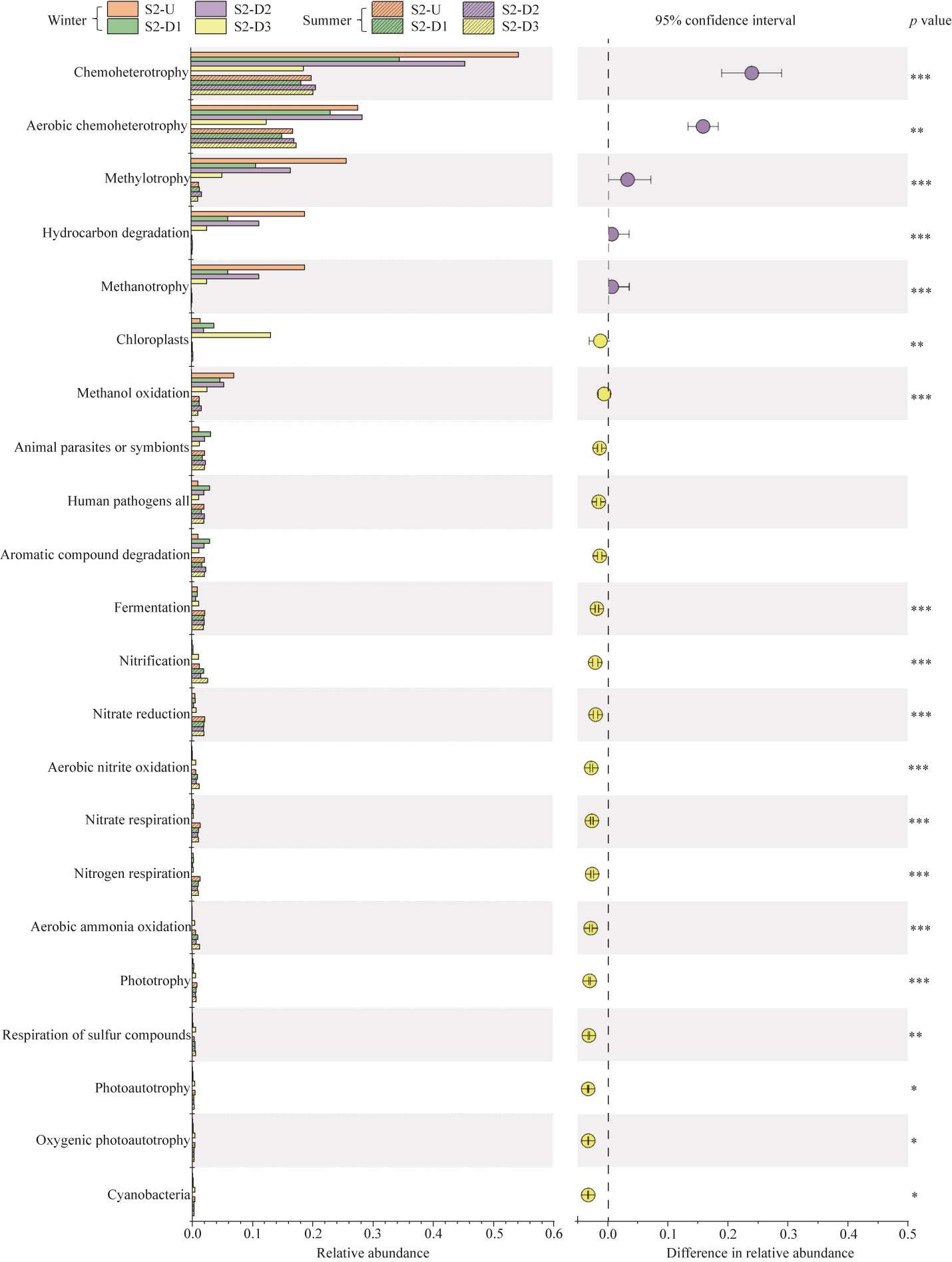


**(b) Sediment**


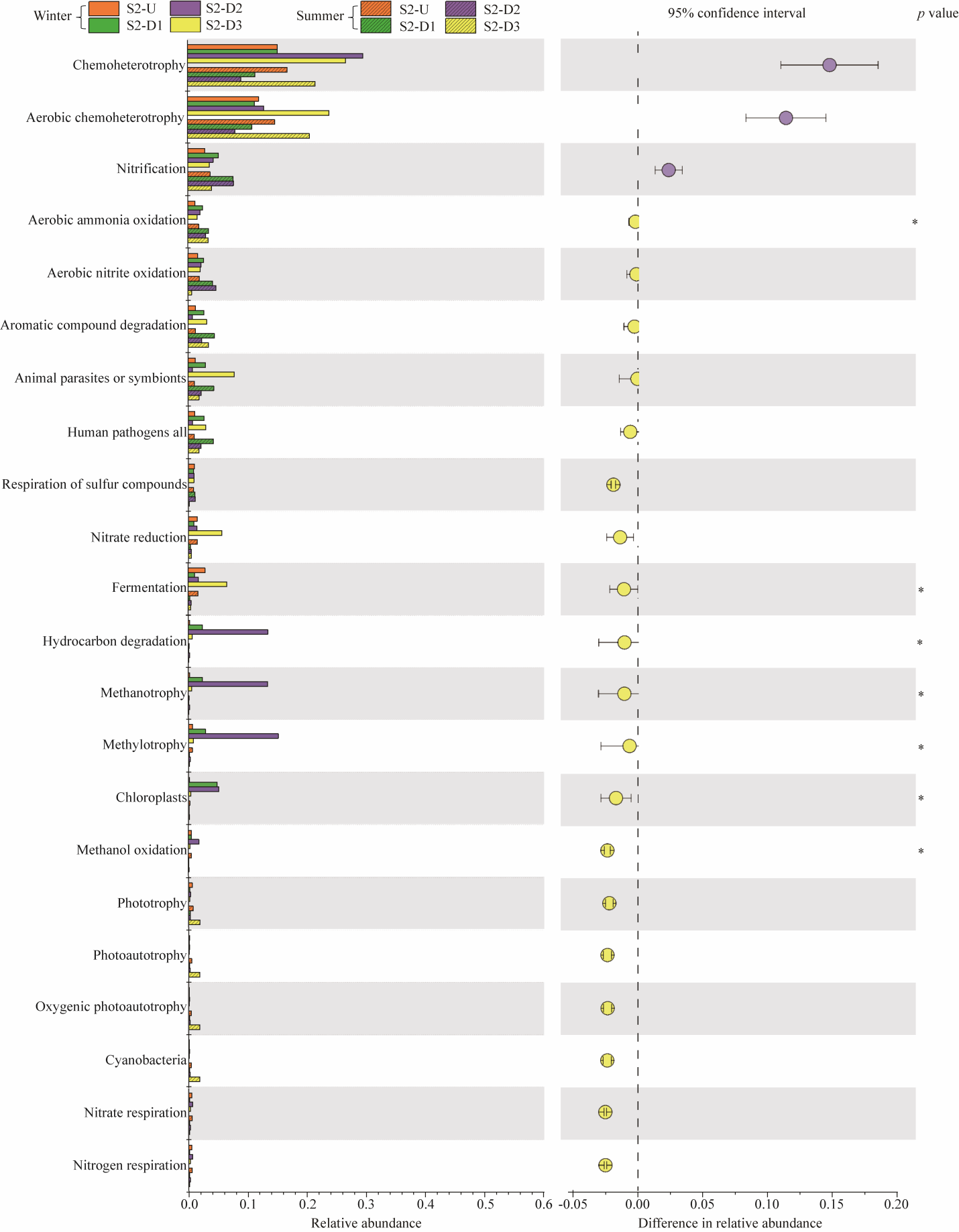


**Fig. S3**. Clustered bar-plots of relative OTU functional group relative abundances based on FAPROTAX in water and sediment from upstream to downstream of S2 in winter (a) and summer (b). The left panel displayed the abundance ratio of different functional groups; the middle showed the percentage of functional group abundance within the 95% confidence interval; the right indicated *p* value. **p <* 0.05, ***p* < 0.01, and ****p* < 0.001.

| **Table S1.** The six cascade hydropower dams constructed and operated in the middle-lower reaches of the Lancang River. | | | | | | |
| --- | --- | --- | --- | --- | --- | --- |
| Name | Normal water level (m) | Dam height (m) | Installed capacity (MW) | Water discharge (m^3^/s) | Reservoir area (km^2^) | Operation year |
| Gongguoqiao (S2) | 1307 | 105 | 900 | 1010 | 16.72 | 2013 |
| Xiaowan (S3) | 1240 | 294.5 | 4200 | 1230 | 189.10 | 2010 |
| Manwan (S4) | 994 | 132 | 1500 | 1236 | 23.60 | 1993 |
| Dachaoshan (S5) | 899 | 115 | 1350 | 1340 | 26.25 | 2003 |
| Nuozhadu (S6) | 812 | 254 | 5850 | 1750 | 320.00 | 2014 |
| Jinghong (S7) | 602 | 108 | 1750 | 1830 | 32.80 | 2009 |

**Table S2**

Physicochemical properties (mean ± standard deviation) of (a) water and (b) sediment samples during winter and summer season.

(a) Water

| Season | Sample | T | pH | ORP | COND | TURB | DO | TDS | TN | TP | N:P |
| --- | --- | --- | --- | --- | --- | --- | --- | --- | --- | --- | --- |
|  |  | °C |  | mV | mS/m | NTU | mg/L | g/L | mg/L | mg/L |  |
| Winter | S1 | 7.59 ± 0.08 | 8.27 ± 0.01 | 235.5 ± 0.71 | 53.50 ± 0.00 | 74.05 ± 0.07 | 18.38 ± 0.10 | 0.34 ± 0.00 | 0.45 ± 0.05 | 0.10 ± 0.01 | 4.56 ± 0.21 |
|  | S2-U | 10.86 ± 0.04 | 7.93 ± 0.32 | 244.00 ± 0.00 | 47.30 ± 1.41 | 93.25 ± 1.20 | 16.55 ± 0.53 | 0.32 ± 0.00 | 1.55 ± 0.10 | 0.02 ± 0.00 | 64.81 ± 11.52 |
|  | S2-D1 | 12.20 ± 1.12 | 7.97 ± 0.11 | 200.50 ± 2.12 | 48.40 ± 0.14 | 86.70 ± 1.13 | 18.47 ± 2.06 | 0.31 ± 0.00 | 1.50 ± 0.11 | 0.03 ± 0.01 | 46.18 ± 11.77 |
|  | S2-D2 | 10.55 ± 0.63 | 8.12 ± 0.02 | 223.50 ± 2.12 | 50.85 ± 0.49 | 103.30 ± 9.48 | 14.53 ± 0.31 | 0.33 ± 0.00 | 1.69 ± 0.03 | 0.03 ± 0.00 | 65.04 ± 1.15 |
|  | S2-D3 | 13.18 ± 0.10 | 8.09 ± 0.08 | 196.50 ± 16.26 | 41.75 ± 0.07 | 10627.50 ± 498.51 | 10.59 ± 0.51 | 0.27 ± 0.00 | 1.49 ± 0.14 | 0.10 ± 0.02 | 14.76 ± 1.42 |
|  | S3-U | 18.33 ± 0.15 | 7.78 ± 0.56 | 227.50 ± 0.71 | 29.10 ± 0.14 | 71.80 ± 4.53 | 9.58 ± 0.42 | 0.19 ± 0.00 | 1.73 ± 0.02 | 0.26 ± 0.05 | 6.78 ± 1.36 |
|  | S3-D | 16.38 ± 0.01 | 8.10 ± 0.06 | 216.50 ± 3.54 | 36.25 ± 0.07 | 45.85 ± 0.07 | 10.03 ± 0.40 | 0.24 ± 0.00 | 1.97 ± 0.16 | 0.26 ± 0.00 | 7.56 ± 061 |
|  | S4-D | 15.30 ± 0.41 | 8.10 ± 0.32 | 244.00 ± 22.63 | 37.15 ± 0.07 | 49.35 ± 0.92 | 12.40 ± 0.14 | 0.24 ± 0.00 | 0.57 ± 0.02 | 0.02 ± 0.00 | 24.74 ± 2.29 |
|  | S5-D | 17.39 ± 0.55 | 7.97 ± 0.06 | 248.00 ± 2.83 | 36.40 ± 0.28 | 63.50 ± 7.50 | 12.29 ± 0.30 | 0.24 ± 0.00 | 0.60 ± 0.02 | 0.06 ± 0.00 | 9.77 ± 0.80 |
|  | S6-U | 19.82 ± 0.30 | 7.12 ± 0.04 | 271.00 ± 1.41 | 24.35 ± 0.21 | 44.10 ± 0.14 | 12.28 ± 1.75 | 0.16 ± 0.00 | 0.58 ± 0.01 | 0.00 ± 0.00 | 169.58 ± 38.30 |
|  | S6-D | 18.73 ± 0.11 | 7.15 ± 0.04 | 281.50 ± 0.71 | 31.35 ± 0.07 | 43.30 ± 0.14 | 12.22 ± 0.12 | 0.20 ± 0.00 | 0.89 ± 0.01 | 0.01 ± 0.00 | 105.00 ± 7.07 |
|  | S7-D | 20.92 ± 0.44 | 7.21 ± 0.06 | 306.00 ± 1.41 | 29.30 ± 0.00 | 71.90 ± 0.57 | 11.81 ± 0.36 | 0.19 ± 0.00 | 0.80 ± 0.00 | 0.02 ± 0.00 | 42.33 ± 10.49 |
|  | S8 | 19.34 ± 0.10 | 7.80 ± 0.03 | 245.00 ± 4.24 | 28.75 ± 0.07 | 100.20 ± 12.45 | 12.64 ± 0.91 | 0.19 ± 0.00 | 0.49 ± 0.01 | 0.01 ± 0.00 | 72.10 ± 16.59 |
| Summer | S2-U | 19.16 ± 0.53 | 6.50 ± 0.02 | 360.00 ± 0.00 | 32.10 ± 0.26 | 625.67 ± 149.58 | 3.98 ± 0.06 | 0.21 ± 0.00 | 0.44 ± 0.13 | 0.10 ± 0.02 | 4.44 ± 1.24 |
|  | S2-D1 | 20.35 ± 2.15 | 7.55 ± 0.11 | 289.00 ± 6.56 | 32.47 ± 0.42 | 1715.00 ± 17.32 | 4.12 ± 0.38 | 0.21 ± 0.00 | 0.25 ± 0.01 | 0.10 ± 0.08 | 3.70 ± 2.03 |
|  | S2-D2 | 20.01 ± 1.94 | 7.65 ± 0.01 | 286.00 ± 0.00 | 31.83 ± 0.78 | 2865.00 ± 93.97 | 5.41 ± 0.15 | 0.21 ± 0.01 | 0.39 ± 0.22 | 0.06 ± 0.01 | 5.88 ± 3.15 |
|  | S2-D3 | 19.22 ± 0.68 | 6.43 ± 0.04 | 347.33 ± 1.53 | 31.07 ± 0.32 | 1815.56 ± 10.18 | 3.29 ± 0.07 | 0.20 ± 0.00 | 0.33 ± 0.09 | 0.24 ± 0.01 | 1.38 ± 0.40 |

(b) Sediment

| Season | Sample | WC | OM | TN | TP | N:P | MA |
| --- | --- | --- | --- | --- | --- | --- | --- |
|  |  | (%) | (%) | mg/L | mg/L |  | µg FDA·g^-1^ sample DW·h^-1^ |
| Winter | S2-U | 50.84 ± 2.85 | 2.74 ± 0.14 | 5.36 ± 2.10 | 4.33 ± 0.02 | 1.29 ± 2.30 | 2.54 ± 0.06 |
|  | S2-D1 | 79.44 ± 0.65 | 0.84 ± 0.10 | 4.84 ± 1.00 | 2.50 ± 0.00 | 1.94 ± 1.89 | 1.98 ± 0.19 |
|  | S2-D2 | 68.97 ± 0.07 | 1.44 ± 0.03 | 4.99 ± 0.03 | 2.50 ± 0.08 | 2.00 ± 1.11 | 1.57 ± 0.17 |
|  | S2-D3 | 65.77 ± 0.17 | 3.64 ± 0.26 | 5.75 ± 0.33 | 3.92 ± 0.11 | 1.46 ± 0.28 | 3.82 ± 0.18 |
| Summer | S2-U | 56.12 ± 4.04 | 2.22 ± 3.48 | 2.41 ± 0.33 | 0.89 ± 1.15 | 2.67 ± 0.42 | 0.59 ± 0.00 |
|  | S2-D1 | 79.82 ± 4.64 | 1.16 ± 0.16 | 1.73 ± 0.24 | 0.53 ± 0.07 | 3.26 ± 0.04 | 0.83 ± 0.06 |
|  | S2-D2 | 76.81 ± 3.57 | 0.85 ± 0.25 | 5.16 ± 0.23 | 0.63 ± 0.08 | 8.28 ± 0.15 | 0.56 ± 0.27 |
|  | S2-D3 | 66.93 ± 0.36 | 1.53 ± 2.03 | 4.13 ± 0.07 | 0.82 ± 0.06 | 5.06 ± 0.01 | 0.63 ± 0.93 |
